# Validation of a DKK1 RNAscope chromogenic in situ hybridization assay for gastric and gastroesophageal junction adenocarcinoma tumors

**DOI:** 10.1038/s41598-021-89060-3

**Published:** 2021-05-10

**Authors:** Charles Caldwell, James B. Rottman, Will Paces, Elizabeth Bueche, Sofia Reitsma, Joseph Gibb, Vitria Adisetiyo, Michael S. Haas, Heidi Heath, Walter Newman, Jason Baum, Roberto Gianani, Michael H. Kagey

**Affiliations:** 1 Flagship Biosciences, Westminster, CO USA; 2 Athenaeum Pathology Consulting, Sudbury, MA USA; 3 Leap Therapeutics Inc., 47 Thorndike Street, Suite B1-1, Cambridge, MA 02141 USA

**Keywords:** Prognostic markers, Tumour biomarkers, RNA, Prognostic markers, Gastric cancer, RNA probes

## Abstract

Dickkopf-1 (DKK1) is a secreted modulator of Wnt signaling that is frequently overexpressed in tumors and associated with poor clinical outcomes. DKN-01 is a humanized monoclonal therapeutic antibody that binds DKK1 with high affinity and has demonstrated clinical activity in gastric/gastroesophageal junction (G/GEJ) patients with elevated tumoral expression of DKK1. Here we report on the validation of a DKK1 RNAscope chromogenic in situ hybridization assay to assess DKK1 expression in G/GEJ tumor tissue. To reduce pathologist time, potential pathologist variability from manual scoring and support pathologist decision making, a digital image analysis algorithm that identifies tumor cells and quantifies the DKK1 signal was developed. Following CLIA guidelines the DKK1 RNAscope chromogenic in situ hybridization assay and digital image analysis algorithm were successfully validated for sensitivity, specificity, accuracy, and precision. The DKK1 RNAscope assay in conjunction with the digital image analysis solution is acceptable for prospective screening of G/GEJ adenocarcinoma patients. The work described here will further advance the companion diagnostic development of our DKK1 RNAscope assay and could generally be used as a guide for the validation of RNAscope assays with digital image quantification.

## Introduction

Wnt signaling is a multifaceted pathway that controls embryonic development, adult tissue homeostasis, cell proliferation, survival, and migration^1–3^. The pathway is frequently dysregulated in oncology and has garnered extensive interest as a therapeutic target^4,5^. Dickkopf-1 (DKK1) is a secreted protein that is best characterized as an antagonist of Wnt/β-catenin dependent (canonical) signaling; however, it has also been implicated in activation of Wnt/β-catenin independent (noncanonical) pathways and PI3K/AKT signaling^6–10^. DKK1 expression is elevated in a range of tumor types and this is frequently associated with a poor clinical prognosis^11^. Nonclinical models have demonstrated that DKK1 can promote tumor growth, stimulate angiogenesis, facilitate metastasis, and favor an immunosuppressive tumor microenvironment^11–14^. The oncogenic activity of DKK1 can be blocked by therapeutic intervention in animal tumor models^14–21^. As such, the targeting of DKK1 with DKN-01, a therapeutic IgG4 antibody, is currently under clinical investigation^22–24^.

We hypothesized that tumoral expression of DKK1 is a predictive biomarker of response to DKN-01. Tumor tissue from relapsed refractory gastric/gastroesophageal junction (G/GEJ) patients that received a DKN-01 + pembrolizumab combination treatment as part of a phase 1b/2a study were retrospectively investigated for DKK1 mRNA levels using the RNAscope chromogenic in situ hybridization (CISH) assay^24^. For a subgroup of these patients who had not previously received an anti-PD-1/PD-L1 therapy, elevated DKK1 tumoral expression was associated with a clinical response and increased progression free survival^24^. For a majority of the tumor tissues, DKK1 tumoral expression was quantified digitally by morphometric analysis and an H-score was calculated by determining the percentages of tumor cells expressing low, medium, and high levels of DKK1^24^. Patients with an H-score ≥ 35, corresponding to the upper tertile of DKK1 expression, were more likely to derive benefit from the DKN-01 + pembrolizumab combination therapy, supporting the notion that elevated tumoral DKK1 expression is a biomarker for G/GEJ patients^24^.

RNAscope is an in situ hybridization technique that has been fully optimized on multiple autostainer platforms. It is a specific and sensitive assay that has been increasingly utilized in numerous research and clinical studies, including the prospective identification of fibroblast growth factor receptor (FGFR) tumor positive patients for treatment with the oral pan-FGFR inhibitor rogaratinib^25–27^. RNAscope probes are bioinformatically designed to selectively target a RNA and can overcome sensitivity and specificity limitations that are frequently encountered with antibodies used for immunohistochemistry (IHC)^25^. Detection of partially degraded RNA, a concern of routine formalin fixed paraffin embedded (FFPE) clinical specimens, is possible because a pool of probes are used which allow for the detection of fragmented RNA^25^. RNAscope is exquisitely sensitive; the signal amplification chemistry allows for detection of a single RNA molecule per cell^25^. Staining appears as dots in the cell with each dot corresponding to a single RNA molecule^25^. The nature of this signal allows for manual semi-quantification by a pathologist or quantification by digital approaches, both involving counting the number of dots per cell. Potential advantages of digital signal quantification are the improved precision, accuracy, and removal of pathologist bias that can occur with manual scoring^28–31^. Additionally, pathologist time can be more efficiently utilized since digital scoring may be performed by trained technicians and reviewed by the pathologist. In summary, RNAscope has clear advantages as a research tool and a clinical diagnostic, especially in circumstances where suitable IHC reagents are not available.

Here we report on the validation of a DKK1 RNAscope CISH assay and a digital image analysis solution to quantify DKK1 tumoral expression according to Clinical Laboratory Improvement Amendments (CLIA) guidelines^32^. We demonstrate that the DKK1 RNAscope assay and digital algorithm are sensitive, specific, accurate, and precise. The digital algorithm is intended for clinical use as a pathologist decision support tool. The results of the digital analysis are reviewed for acceptance by the pathologist prior to reporting data. The validated assay is being applied to prospectively identify G/GEJ adenocarcinoma second-line patients with high tumoral expression of DKK1 for a phase 2 clinical trial in combination with DKN-01 and the anti-PD-1 therapeutic, tislelizumab, and as a retrospective analysis of first-line patients treated with DKN-01 in combination with tislelizumab and chemotherapy (NCT04363801).

## Results

### Initial assessment of a DKK1 RNAscope CISH assay

**Figure 1:**
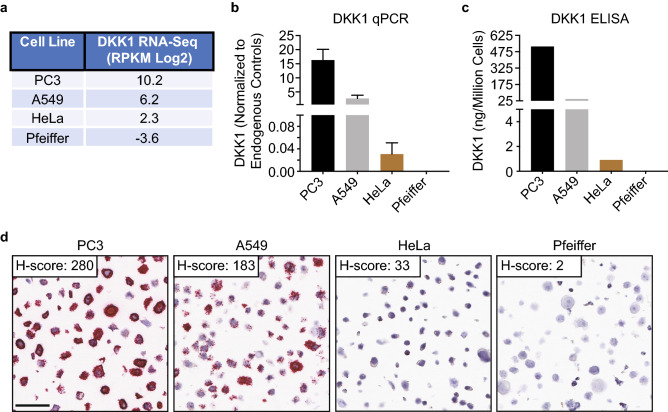
Initial assessment of the DKK1 RNAscope assay. (**a**) Four cell lines that express a dynamic range of DKK1 from very high to none were identified using RNA-Seq data from the Cancer Cell Line Encyclopedia database. Reads per kilobase per million mapped reads (RPKM). (**b**, **c**) DKK1 expression in the indicated cell lines was verified by qPCR and ELISA. DKK1 qPCR data was normalized to GAPDH, TBP, and SDHA housekeeping genes. (**d**) DKK1 RNAscope staining of the control FFPE cell pellet array containing the indicated cell lines. Red dots are indicative of DKK1 signal with each dot corresponding to a single RNA molecule. DKK1 signal was quantified using QuPath morphometric software and an H-score (range 0–300) was calculated as described in the methods. Scale bar: 50 μm.

In order to initially assess a DKK1 RNAscope CISH assay, 4 cell lines (PC3, A549, HeLa, and Pfeiffer) that express a range of DKK1 were identified using publicly available RNA-Seq data from the Cancer Cell Line Encyclopedia (CCLE) database (Fig. 1a and Supplementary Table S1)^33^. The CCLE DKK1 RNA-Seq data for the 4 cell lines was confirmed by quantitative polymerase chain reaction (qPCR) and an enzyme-linked immunosorbent assay (ELISA) (Fig. 1b, c). A control FFPE cell pellet array (CPA) consisting of the 4 cell lines was generated and assessed by RNAscope for RNA integrity and background signal with probes to the moderately expressed reference gene peptidylprolyl isomerase B (PPIB) and to the bacterial gene dihydrodipicolinate reductase (dapB), respectively (Supplementary Fig. S1). All 4 cell pellets demonstrated robust PPIB signal and no detectable dapB signal. The expected DKK1 RNAscope staining pattern was observed with the strongest signal occurring in PC3 cells, reduced signal in A549 and HeLa cells, and absent signal in the Pfeiffer cells (Fig. 1d). The open source software program QuPath was used to quantify the amount of DKK1 signal and the digital H-scores confirmed the observed staining^34^. These data indicate that the RNAscope assay can detect DKK1 expression over a wide dynamic range and that the assay is likely specific given that no detectable signal occurred in the Pfeiffer cells.

**Figure 2:**
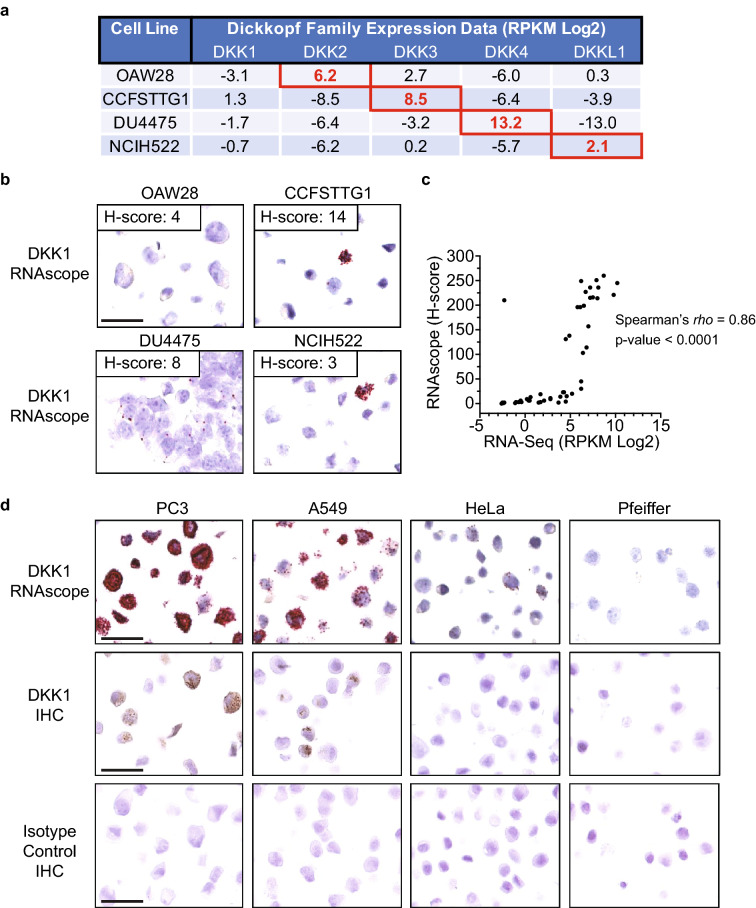
Specificity and accuracy of the DKK1 RNAscope assay. (**a**) Cell lines were identified using RNA-Seq data from the Cancer Cell Line Encyclopedia database that expressed very high levels of the indicated Dickkopf family member and low levels of DKK1. Red boxes denote the expression level of the Dickkopf family member that is highly expressed in the indicated cell line. Reads per kilobase per million mapped reads (RPKM). (**b**) The specificity FFPE cell pellet array with the indicated cell lines was stained for DKK1, quantified using QuPath morphometric software and an H-score (range 0–300) was calculated as described in the methods. Scale bar: 50 μm. (**c**) FFPE cell pellet arrays with different cancer cell lines were stained for DKK1, quantified using QuPath morphometric software and an H-score (range 0–300) was calculated as described in the methods. DKK1 RNA-Seq data was obtained from the Cancer Cell Line Encyclopedia database. Reads per kilobase per million mapped reads (RPKM). (**d**) The indicated cells were stained for DKK1 RNA by RNAscope (top row), DKK1 protein by IHC (middle row) or an isotype control antibody (bottom row). Scale bar: 50 μm.

In order to initially assess specificity of the DKK1 RNAscope assay, a specificity FFPE CPA was generated with cell lines that express high levels of other Dickkopf family members (DKK2, DKK3, DKK4 or DKKL1) and low levels of DKK1. These cell lines were selected using the CCLE database and verified by qPCR (Fig. 2a, Supplementary Fig. S2a and Supplementary Table S1)^33^. All cell pellets had robust PPIB signal and no detectable dapB signal (Supplementary Fig. S2b). Very little DKK1 signal was observed in the specificity CPA indicating that the DKK1 RNAscope probes are not cross reacting with other Dickkopf family member RNA (Fig. 2b). The DKK1 signal that was observed in a small number of cells is explained by the low level of DKK1 expression. To further assess specificity and accuracy, two FFPE CPAs consisting of different cancer cell lines were stained for DKK1 (Fig. 2c and Supplementary Table S2). QuPath digital H-scores were determined for cell pellets that exhibited acceptable RNA integrity (PPIB staining) and background (dapB staining). H-scores were compared to the 48 cell lines with available DKK1 RNA-Seq data from the CCLE database and a significant correlation was observed (Spearman’s *rho* = 0.86, *p* value < 0.0001) supporting the specificity and accuracy of the DKK1 RNAscope assay^33^. Accuracy was further assessed by comparing the DKK1 RNAscope assay to a DKK1 IHC assay (Fig. 2d). The RNAscope and IHC data were largely consistent across the control CPA, with both assays demonstrating the most robust signal in PC3 cells and a lack of signal in Pfeiffer cells. However, the RNAscope assay is much more sensitive and was able to detect RNA in the HeLa cell pellet whereas no IHC signal was observed. Taken together, the consistency of the DKK1 RNAscope results with the qPCR, ELISA, RNA-Seq and IHC data across multiple different cancer cell lines indicates that the DKK1 RNAscope assay is highly specific and accurate.

### Validation of the DKK1 RNAscope CISH assay

Validation of the DKK1 RNAscope CISH assay was conducted to assess specificity, sensitivity, accuracy, and precision in G/GEJ tumor resections according to CLIA guidelines. Briefly, 40 G/GEJ tumor resections were assessed, and the CISH assay passed the pre-defined acceptance criteria for specificity, sensitivity, accuracy, and precision (Table 1). The same lot of probes were used during the course of the study. The details of the validation are summarized below.

**Table 1:** DKK1 RNAscope assay validation results.

Performance parameter^a^	Tumor resections evaluated (n)	Percentage pass	Parameter pass/fail
Analytical specificity^b^	40	100% (40/40)	Pass
Analytical sensitivity^c^	40	100% (40/40)	Pass
Accuracy^d^	20	*rho* = 0.629, *p* value = 0.003	Pass
Precision^e^	12	92% (11/12)	Pass

^a^Details of the pre-defined acceptance criteria are described in the methods.

^b^Signal was predominantly localized to tumor cells.

^c^Signal was detected above background.

^d^Spearman correlation (*p* value < 0.05) with DKK1 qPCR results.

^e^Results across 3 separate staining days within the same binned category of expression (negative bin: H-score = 0, low bin: H-score < 34, and high bin: H-score ≥ 35). In cases of discordant binning of negative, low or high categories, a ± 20 point H-score discrepancy was still considered acceptable.

**Figure 3:**
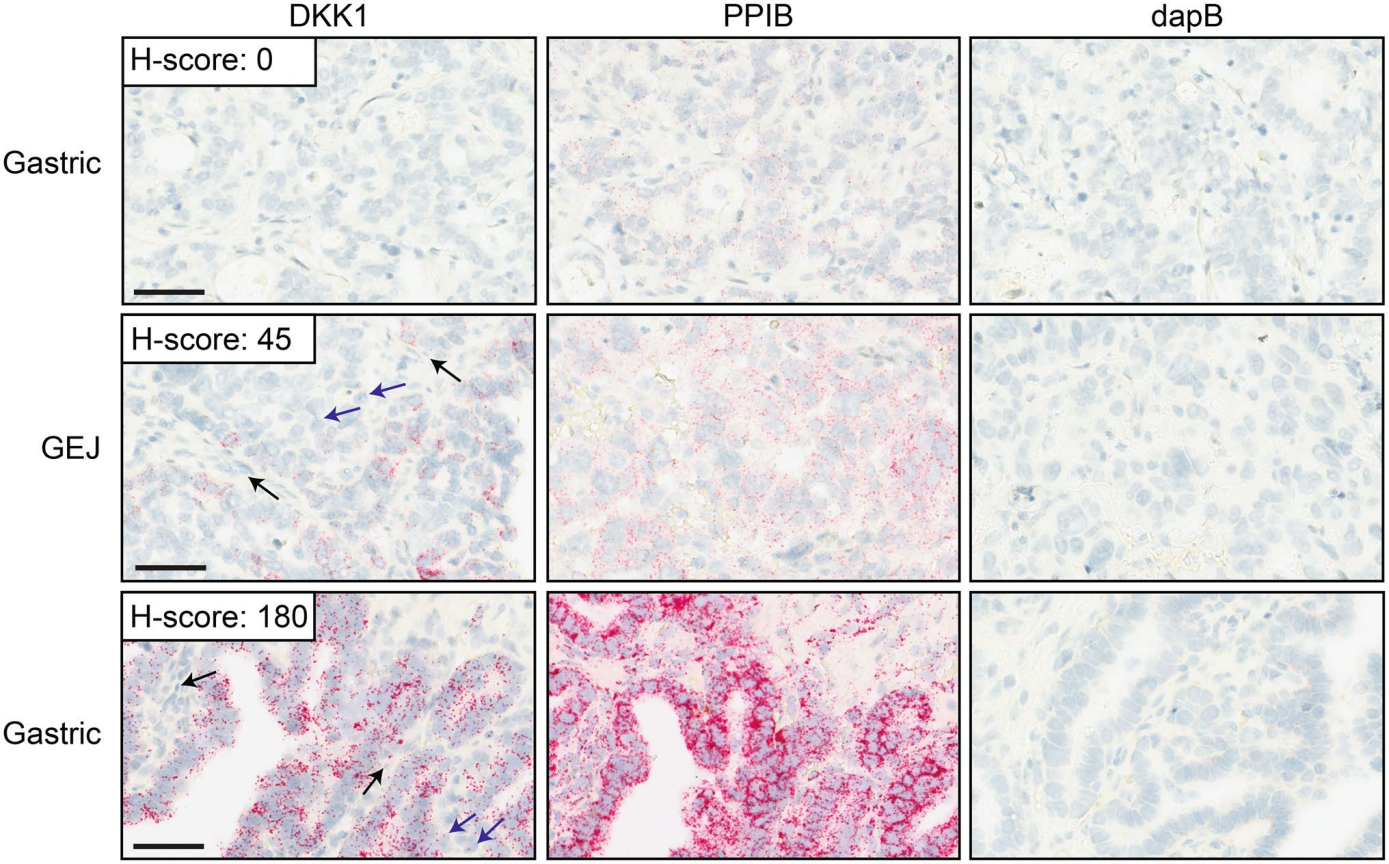
The DKK1 RNAscope assay in tumor resections. Representative images from 3 tumor resections with no (top row), moderate (middle row) and high (bottom row) DKK1 signal are shown with the PPIB control for RNA integrity and the dapB background control. DKK1 H-scores (range 0–300) were semi-quantified manually as described in the methods. Black arrows denotes non tumoral cells without DKK1 signal. Blue arrows denote tumor cells with low levels of DKK1 signal (one dot per cell). Scale bar: 50 μm.

All tumor resections had adequate RNA integrity and acceptable background as determined by presence of PPIB signal and absence of dapB signal, respectively (Supplementary Table S3). A region of analysis (ROA) that contained viable tumor cells and sufficient PPIB signal (≥ 4 dots/cell) was annotated for DKK1 scoring and manually assigned an H-score for the tumor cells. A dynamic range of DKK1 signal (H-scores of 0–180) was observed in the tumor cells (Supplementary Table S3). Signal was localized to tumor tissue and was rarely detected in non tumoral cells, demonstrating specificity of the assay (Fig. 3). Sensitivity of the assay was confirmed by detecting tumor cells with a range of DKK1 expression, including cells with only a single dot which corresponds to one molecule of RNA^25^. For some tumor resections, less intense DKK1 and PPIB signal was observed. This is a reflection of partial RNA degradation resulting in the hybridization of fewer RNAscope probes. Since the number of dots and not the intensity is used to assess the DKK1 signal, samples with RNA degradation that still had sufficient PPIB signal (≥ 4 dots/cell) were acceptable for evaluation.

Orthogonal confirmation of the DKK1 RNAscope assay’s accuracy was assessed by qPCR. A statistically significant Spearman correlation (*rho* = 0.63, *p* value = 0.003) to the manual H-score data was obtained (Supplementary Fig. S3a). The correlation was the same regardless if the qPCR normalization was conducted with RPLP0 or SDHA housekeeping genes. It should be noted that some outlier values were observed. Possible reasons to explain discrepancies include challenges in isolating pure tumoral tissue for RNA extraction and qPCR analysis, biologic variation in cellularity from different sections of the tumor resections, inhibitory substances in the tissues that affect the efficiency of the qPCR reaction, and nonspecific qPCR amplification. Precision was evaluated in 12 tumor resections. Three slide sections from each tumor resection were stained on 3 separate days and images were evaluated (Supplementary Fig. S3b and Supplementary Table S4). Eleven out of 12 tumor resections passed the pre-determined criteria for precision acceptance requiring consistent binning of negative (H-score = 0), low (H-score < 34) and high (H-score ≥ 35) DKK1 signal. The rationale for setting a DKK1 H-score threshold of ≥ 35 for high versus low DKK1 was based on a retrospective analysis demonstrating improved clinical benefit in DKN-01 + pembrolizumab treated G/GEJ patients with tumoral expression above this threshold^24^. Taken together these results indicate that the DKK1 RNAscope assay is specific, sensitive, accurate, and precise for staining of G/GEJ tumor tissue.

### Validation of the DKK1 RNAscope digital image analysis solution

**Figure 4:**
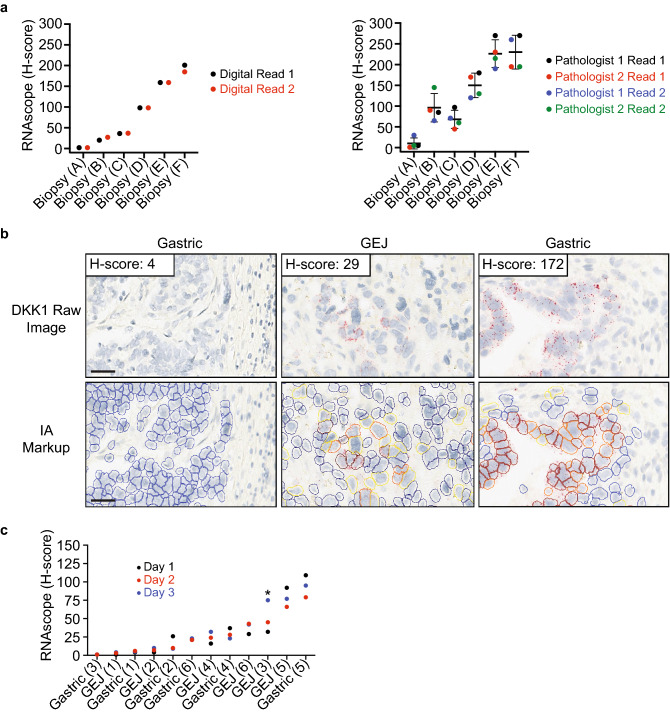
Validation of the digital image analysis algorithm to quantify DKK1 RNAscope signal. (**a**) Six gastric/gastroesophageal junction tumor biopsies with a range of DKK1 expression were stained for DKK1 by RNAscope. Images were scored in duplicate using an image analysis solution from Flagship Biosciences (left graph) and an H-score (range 0–300) was calculated as described in the methods. The same images were scored manually in duplicate with a one week washout period by two separate pathologists (right panel). Mean and standard deviation are shown. (**b**) Representative images from 3 tumor resections with no, low, and high DKK1 signal and the image analysis markup are shown. Markup up colors correspond to the following; blue indicates tumor cells with no DKK1 signal, yellow represents tumors cells with low signal (1–3 dots), orange selected tumors cells have medium signal (4–9 dots), and red identifies tumors cells with high signal (10 + dots). Stromal cells which are not highlighted by the algorithm are not scored. Image analysis (IA). Scale bar: 10 μm. (**c**) Images from replicate DKK1 staining conducted on 3 different days were analyzed and H-scores were quantified. All replicates were required to be in the same bin category or within an H-score of ± 20 if discordant binning occurred. Bins were defined as, negative signal (H-score = 0), low signal (H-score < 34), and high signal (H-score ≥ 35). Asterisk indicates a resection that did not meet the precision criteria.

Manually scoring RNAscope stained slides can be challenging since it requires the pathologist to track multiple pieces of information over potentially hundreds of thousands of cells, such as dots per cell, the number of cells binned as negative, low, medium, and high signal, and consideration of which cells are tumor cells. High levels of magnification are frequently required to accurately assess the number of dots per cell, which can result in whole tissue context being lost and inconsistent results. Digital pathology can address these challenges by reducing variability and subjectivity through establishing a consistent cell identification and scoring algorithm. RNAscope is well suited for digital pathology given the nature of the signal, dots per cell, that can be quantified unbiasedly by software. We developed an image analysis algorithm to identify tumor cells, quantify the amount of DKK1 signal by numbers of dots per cell, and finally calculate a digital H-score. In order to initially assess a digital approach, 6 G/GEJ tumor tissues with a range of DKK1 expression, were digitally scored in duplicate and compared to manual duplicate scoring by two separate pathologists (Fig. 4a). These results clearly demonstrate a much lower degree of variability when using the digital scoring algorithm, and thereby support a digital image analysis solution for quantifying DKK1 RNAscope signal in G/GEJ tumor tissue. Given the encouraging initial results, validation of the digital image analysis solution was conducted following CLIA guidelines with pre-defined acceptance criteria to assess the specificity, sensitivity, accuracy, and precision of the algorithm. Scanned 40 × slide images from the DKK1 RNAscope assay validation were used and the digital image analysis algorithm was successfully validated (Table 2). The details of the validation are summarized below.

**Table 2:** DKK1 RNAscope digital image analysis validation results.

Performance parameter^a^	Tumor resections evaluated (n)	Percentage pass	Parameter pass/fail
Analytical specificity^b^	40	90% (36/40)	Pass
Analytical sensitivity^c^	40	100% (40/40)	Pass
Accuracy^d^	36	*r* = 0.74, *p* value < 0.0001	Pass
Precision^e^	12	92% (11/12)	Pass

^a^Details of the pre-defined acceptance criteria are described in the methods.

^b^Algorithm accurately identified cells as objects, accurately classified tumor and stromal compartments of the tissue, and accurately classified DKK1 negative cells.

^c^Algorithm accurately identified cells when present and classified DKK1 positive cells correctly.

^d^Pearson correlation (*p* value < 0.05) with DKK1 manual H-scores.

^e^Results across 3 separate staining days within the same binned category of expression (negative bin: H-score = 0, low bin: H-score < 34, and high bin: H-score ≥ 35). In cases of discordant binning of negative, low or high categories, a ± 20 point H-score discrepancy was still considered acceptable.

The algorithm was able to successfully identify tumor cells from non-tumor cells and categorize cells as either positive or negative for DKK1 signal, thereby passing sensitivity and specificity (Fig. 4b). All tumor resections passed the specificity assessment, with the exception of 4 samples (Supplementary Table S5). The failures occurred from the algorithm detecting background signal resulting from endogenous alkaline phosphatase activity. While this is expected to be a rare occurrence, background signal from endogenous alkaline phosphatase activity is visibly distinct from true DKK1 signal and in practice will be flagged by the scoring pathologist. Accuracy was assessed by comparing the manual and digital H-scores after removing the 4 tumor resections that failed specificity (Supplementary Fig. S4). A statistically significant correlation was observed (Pearson’s *r* = 0.74, *p* value < 0.001). There was a general trend for higher manual H-scores, which was attributed to the challenges of manually scoring these large tumor resections which on average contained approximately 300,000 cells detected by image analysis (Supplementary Table S5). Precision was evaluated in 12 tumor resections. Three slide sections from each tumor resection were stained on 3 separate days and images were evaluated (Fig. 4c and Supplementary Table S6). Eleven out of 12 tumor resections passed the pre-determined criteria for acceptance. The 3 replicates were required to fall within a negative signal (H-score = 0), low signal (H-score < 34) or high signal (H-score ≥ 35) bin category or if discordant binning occurred then to be within an H-score of ± 20. The cutoff of 35 was established from a retrospective analysis demonstrating that G/GEJ patients with elevated tumoral expression in the upper tertile (H-score ≥ 35) experienced clinical benefit to a DKN-01 + pembrolizumab combination therapy^24^. The resection that failed precision had H-scores ranging from 32 to 75. A closer inspection revealed that the variance for this resection could likely be explained by the manual annotation of the ROA not being exactly the same for all 3 replicates, and therefore was not a reflection of variance from the digital algorithm. Taken together, these results indicate that the digital image solution is specific, sensitive, accurate, and precise for DKK1 RNAscope staining of G/GEJ tumor tissue.

## Discussion

Biomarkers have had increasing prominence in drug development and are often applied in clinical trial design to enrich for patients most likely to derive benefit from a specific therapeutic or treatment regimen. In order to use a biomarker strategy to prospectively identify patients for a therapy, it is imperative that the biomarker test is robust and precise. Here we report on the successful validation of a G/GEJ tumoral DKK1 RNAscope assay following CLIA guidelines. Because of the challenges in manual semi-quantification of RNAscope staining, we developed a novel digital image analysis algorithm that is sensitive, specific, accurate, and precise. The DKK1 RNAscope laboratory developed test is currently being applied as part of a phase 2 clinical study of DKN-01 in combination with tislelizumab to prospectively identify previously treated G/GEJ adenocarcinoma patients with elevated DKK1 tumoral expression, and as a retrospective analysis of first-line patients treated with DKN-01 in combination with tislelizumab and chemotherapy (NCT04363801). To our knowledge this is the first example of an RNAscope assay using a digital image analysis solution for patient enrollment.

RNAscope has been gaining in prominence and has several advantages as a technique for biomarker assessment and development, especially in circumstances where suitable IHC reagents are not available^35–38^. It is highly specific with probes that are computationally designed to decrease the risk of cross reactivity to non-target RNAs, and extremely sensitive by detecting a single RNA per cell^25^. Our CPA data demonstrated specificity and sensitivity with DKK1 signal levels that correlated well with orthogonal methods and no evidence for cross reactivity with other Dickkopf family members. As part of the formal validation following CLIA guidelines, DKK1 staining was shown to be localized to tumor cells in G/GEJ resections with a wide dynamic signal range, therefore demonstrating specificity and sensitivity. Accuracy of the DKK1 RNAscope assay was first assessed in CPAs using four different orthogonal methods: qPCR, RNA-Seq, ELISA, and IHC. In all instances there was correlation with the DKK1 RNAscope data, strongly supporting the accuracy of the assay. For the formal accuracy assessment following CLIA guidelines, DKK1 qPCR data was generated and compared to the manual H-score data for 20 G/GEJ tumor resections. While a statistically significant correlation occurred between the H-score data and the qPCR results, there were examples of nonconcordant samples. We believe this is a reflection of the inherent challenges of measuring DKK1 expression by qPCR from FFPE tumor resections and not a reflection of the assay. Finally, precision was assessed on sections from G/GEJ tumor resections stained on 3 separate days using the fully automated Leica Bond platform to reduce the risk of technical variability. Of the 12 tumor resections analyzed, 11 passed the pre-defined criteria for manual pathology scoring, thereby demonstrating precision. The RNAscope technique has clear advantages for biomarker assessment and development, and the DKK1 RNAscope assay is sensitive, specific, accurate, and precise.

A potential challenge with RNAscope is manual scoring of the signal. This is especially true for an H-score which requires the pathologist to score the tissue at a high magnification in order to visualize the number of dots in the tumor cells. This can cause the pathologist to lose the context of the whole tissue leading to inaccurate and non-reproducible scoring, which is especially problematic for larger tissues. We first explored this issue using a set of 6 G/GEJ tumor tissues with a range of DKK1 expression. We observed both inter- and intra-pathologist manual scoring variability, which was greatly reduced when a digital image analysis algorithm was applied. Given this encouraging initial data, we undertook a formal evaluation of the sensitivity, specificity, accuracy, and precision of a digital algorithm following CLIA guidelines. The algorithm performed well for both sensitivity and specificity by accurately identifying tumor cells and correctly classifying tumor cells as positive or negative for DKK1 signal. However, 4 tumor resections failed for specificity because of background signal from endogenous alkaline phosphatase. In clinical practice the integration of pathologist review is a part of the total assay workflow and therefore these samples would be flagged and reverted to a manual review. Even in samples which do not pass the criteria for reporting a digital score, the digital solution still serves as a pathologist support tool, to better inform the pathologist’s manual score. The digital algorithm passed formal validation for accuracy by demonstrating a significant correlation with the manual H-score data. There was some variability with a trend for higher manual H-scores, but as discussed, we believe this discrepancy is a reflection of the challenges of manual scoring, especially for the large tumor resections used in this study. When large tumor resections which contain hundreds of thousands to millions of cells are considered, the pathologist may have a tendency to accurately score positive cells but misrepresent the number of negative cells to be counted, referred to in this study as ‘the denominator effect’. Image analysis will reliably count every cell in the tissue and attribute scoring in the same way each time the algorithm is run. Finally, the precision of the digital algorithm was demonstrated by scoring sections that were stained on 3 separate days. Eleven out of the 12 tested tumor resections passed the pre-defined precision criteria. Variation in signal quantification for the precision study is mostly attributed to the inherent cellular differences between sections and not necessarily the algorithm. The digital image analysis algorithm is robust and, in conjunction with the DKK1 RNAscope assay, can accurately and precisely quantify DKK1 signal in G/GEJ tumors.

In conclusion, the DKK1 RNAscope assay was validated as sensitive, specific, accurate, and precise following CLIA guidelines. To reduce potential pathologist variability from manual scoring and support pathologist decision making, a digital image analysis algorithm that identifies tumor cells and quantifies the amount of DKK1 signal was developed and demonstrated to also be sensitive, specific, accurate, and precise. The DKK1 RNAscope laboratory developed test is currently being applied as part of a phase 2 clinical study of DKN-01 in combination with tislelizumab to prospectively identify second-line G/GEJ adenocarcinoma patients with elevated tumoral expression, and as a retrospective analysis of first-line patients treated with DKN-01 in combination with tislelizumab and chemotherapy (NCT04363801). The work described here will further advance the companion diagnostic development of our DKK1 RNAscope assay and could generally be used as a guide for the validation of RNAscope assays with digital image quantification.

## Methods

### Cell culture and FFPE CPA generation

Cell lines PC3 (ATCC: CRL-1435), A549 (ATCC: CCL-185), HeLa (ATCC: CCL-2), Pfeiffer (ATCC: CRL-2632), OAW28 (Sigma: 85101601), CCFSTTG1 (ATCC: CRL-1718), DU4475 (ATCC: HTB-123), and NCIH522 (ATCC: CRL-5810) were cultured using standard techniques. Adherent cells were removed with PBS + 10 mM EDTA. 5 × 10^7^ cells were centrifuged at 800 g and pellets were resuspended in 10% neutral buffered formalin for 24 h at room temperature. Cells were subsequently centrifuged at 800 g, resuspended in 70% EtOH and stored at 4 °C until embedding into a paraffin block. PC3, A549, HeLa, and Pfeiffer cell pellets were embedded into a single block and designated as the control CPA. OAW28, CCFSTTG1, DU4475, and NCIH522 cell pellets were embedded into a single block and designated as the specificity CPA. The 60 cancer cell line FFPE CPAs were generated by Advanced Cell Diagnostics. Cell lines were fixed in 10% neutral buffered formalin for 24 h at 27 °C and then embedded into a paraffin block.

### qPCR

qPCR from cell lines was conducted by isolating RNA from 10^6^ cells using TRIzol reagent (Invitrogen) and phase lock tubes (Invitrogen), following the manufacture’s protocols. cDNA synthesis was done with a QuantiTect Reverse Transcription Kit (Qiagen) and qPCR was conducted in triplicate with TaqMan Fast Advanced Master Mix (Applied Biosystems) according to the manufacture’s protocols with the following TaqMan assays: DKK1 (Hs00183740_m1), DKK2 (Hs00205294_m1), DKK3 (Hs00247429_m1), DKK4 (Hs00205290_m1), DKKL1 (Hs01011550_g1), GAPDH (Hs03929097_g1), TBP (Hs99999910_m1), and SDHA (Hs00188166_m1). qPCR was run on a LightCycler 480 Instrument (Roche). Normalization was conducted using the delta Ct method and the combined Ct values from three separately run endogenous control genes (GAPDH, TBP and SDHA).

qPCR from tumor resections was performed at Canopy Biosciences (Hayward, CA). FFPE tumor tissue was dissected, deparaffinized and dehydrated through Envirene (Hardy Diagnostics) and absolute ethanol, respectively. The air-dried tissue was scraped from each slide and added to lysis buffer (1% SDS and 25% proteinase K in Roche’s High Pure FFPET Kit Lysis Buffer). Samples were lysed overnight at 55 °C with shaking, and RNA was isolated according to the kit manufacturer’s instructions (High Pure FFPET RNA Isolation Kit, Roche). cDNA synthesis was conducted with a High-Capacity cDNA Reverse Transcription Kit (Applied Biosystems) and qPCR was conducted in triplicate with TaqMan Fast Advanced Master Mix (Applied Biosystems) according to the manufacture’s protocols with the following TaqMan assays: DKK1 (Hs00183740_m1), RPLP0 (Hs00420895_gH), and SDHA (Hs00188166_m1). qPCR was run on a 7500 Real-Time PCR System (Applied Biosystems). Normalization was conducted using the delta Ct method and Ct values from the RPLP0 or SDHA endogenous control genes.

### DKK1 ELISA and IHC

DKK1 ELISAs were conducted using a Human DKK1 ELISA kit (Abcam: ab100501). Supernatants from each of the cell lines were analyzed in duplicate following the manufacturer’s protocol. Assay plates were read at 450 nm using the EnSpire Multimode Reader (PerkinElmer). A standard curve was generated using a four-parameter logistic regression fit. The amount of DKK1 was normalized to viable cell number which was determined prior to harvesting the supernatant. DKK1 IHC was performed at StageBio (Worcester, MA). FFPE CPA slide sections were subjected to high temperature antigen retrieval with DIVA Decloaker (Biocare Medical), followed by endogenous peroxidase blockade with Peroxidazed (Biocare Medical) and nonspecific protein binding blockade with Background Punisher (Biocare Medical). Subsequently, sections were incubated at room temperature for 2 h with an anti-DKK1 antibody (Cell Signaling: 4687) or a rabbit polyclonal IgG (Thermo-Fisher: 02-6102) at a concentration of 1.0 mg/mL. After washing, slides were incubated with a Rabbit-on-Farma HRP-Polymer (Biocare Medical) at room temperature for 30 min, washed, and developed for 5 min with a Betazoid DAB chromogen kit (Biocare Medical).

### RNAscope

G/GEJ FFPE tumor resections were commercially acquired from Avaden Biosciences, Discovery Life Sciences or BioOptions following IRB compliance. RNAscope was performed at Advanced Cell Diagnostics (Newark, CA) or Flagship Biosciences (Westminster, CO). Five μm FFPE sections were evaluated by RNAscope CISH for the expression of the following RNAs using Advanced Cell Diagnostics (ACD) probes specific for DKK1 (421418), PPIB (313908), and bacterial dapB (312038). RNAscope probes were designed by ACD as described^25^. The RNAscope CISH assays were performed using the 2.5 LS (ACD: 322150) or LSx (ACD: 322750) Red Reagent Kit on the Leica Biosystems BOND RX platform according to the automated RNAscope protocol optimized for use on the instrument. FFPE sections were placed on the instrument, deparaffinized, subjected to antigen retrieval using Leica Epitope Retrieval Buffer 2 for 15 min at 88 °C (CPAs) or 90 °C (all tumor tissues except for Fig. 4a which were treated at 95 °C), and then treated with protease III (15 min at 40 °C). Probes were hybridized for 2 h at 42 °C followed by signal amplification. Chromogenic detection was then performed using a Bond Polymer Refine Red Detection kit. The slides were counterstained with hematoxylin on the Leica Bond. Slides were scanned at 40x (Aperio AT Turbo or Aperio AT2). All tumor tissues were required to have sufficiently intact RNA (PPIB of ≥ 4 dots/cell), minimal background signal (dapB of < 1–3 dots/cell), and at least 100 evaluable tumor cells from non necrotic tissue. Unanalyzable tissue due to such factors as necrosis, folding, dust, crush artifacts, or other tissue-specific artifacts were excluded from analysis. For tumor resections used for validation, a board-certified MD pathologist annotated ROAs with sufficient PPIB expression on digital images of the PPIB stained tissue; these ROAs were referenced for the manual and digital scoring assessments of DKK1. An H-score was calculated either manually or digitally by determining the percentage of low (1–3 dots/cell), medium (4–9 dots/cell), and high (10 + dots/cell) DKK1 staining tumor cells and using the following formula: H-score = (%High)*3 + (%Medium)*2 + (%Low)*1. The theoretical maximal H-score is 300.

### Digital pathology

For CPAs, QuPath open-source morphometric analysis software was used to quantify DKK1 signal^34^. Briefly, the software identified individual cells using the hematoxylin stain, counted the number of distinct punctate red dots in each cell, and calculated an H-score as described above. For tumor resections, Flagship Bioscience’s image analysis solution was used to quantify DKK1 signal. DKK1 stained 40 × images were manually annotated for ROAs based on PPIB positivity and verified for accuracy by a board-certified MD pathologist. Flagship Biosciences’ image analysis software identified all cells within the tissue via nucleus recognition artificial intelligence. Identified cells were then measured and associated with large data profiles describing each cell’s morphology, staining, and spatial characteristics. Flagship’s image analysis platform used the DKK1 RNAscope stained image and proprietary machine learning to separate the tumor compartment from the stromal compartment. To provide the machine learning algorithm cellular data to learn from, an analyst circled small areas of tumor and small areas of stroma within each tissue section. The tumor/stroma machine learning algorithm then used cellular data profiles and input ROA information to separate tumor and stroma on the entire slide. All cell recognition, stain recognition, and tumor/stroma separations were reviewed by a board-certified MD pathologist. Flagship’s proprietary algorithms were used to identify cells and classify DKK1 tumor signal as negative (no dots/cell), low (1–3 dots/cell), medium (4–9 dots/cell), and high (10 + dots/cell) in the defined ROAs. An H-score was calculated by the software as described above. All dots had to meet a defined staining intensity threshold that was established to be above background. The threshold was verified by a board-certified MD pathologist who confirmed appropriate algorithm performance in each slide and across the ROA, according to two main criteria: (1) correct identification of cells in the ROA and (2) correct identification and classification of DKK1 staining positivity.

### DKK1 RNAscope assay acceptance criteria

Sensitivity, specificity, accuracy and precision of the DKK1 RNAscope assay were evaluated following CLIA guidelines. Twenty gastric and 20 gastroesophageal junction (GEJ) FFPE tumor resections were used to assess sensitivity and specificity, and 90% of the samples were required to pass the specificity and sensitivity criteria. Specificity was defined as a sample demonstrating appropriate cell type staining to tumor cells with no to minimal staining observed in non tumoral cells. Sensitivity was defined as samples demonstrating a dynamic range of DKK1 signal above background from very low (1 dot/cell) to very high (10 + dots/cell). Accuracy was evaluated using 20 tumor resections (10 gastric and 10 GEJ) and required a positive Spearman correlation with a statistically significant *p* value (≤ 0.05) between DKK1 qPCR data and manual DKK1 H-scores. For precision, 12 tumor resections (6 gastric and 6 GEJ) were tested and at least 80% were required to show acceptable staining for all 3 staining runs conducted on different days. Individual tumor resections were considered acceptable when blinded pathology assessment of staining between different runs yielded results within the same binned category of expression. Bins were defined as, negative signal (H-score = 0), low signal (H-score < 34), and high signal (H-score ≥ 35). In cases of discordant binning of negative, low or high categories, a ± 20 point H-score discrepancy was still considered acceptable.

### Digital image analysis algorithm acceptance criteria

Scanned 40 × images of the 40 DKK1 stained G/GEJ tumor resections were used to assess sensitivity, specificity, accuracy, and precision of the digital image analysis algorithm following CLIA guidelines. 85% of the samples were required to pass the specificity and sensitivity criteria. Specificity was defined as the ability of the algorithm to identify cells as objects only when they corresponded to true cells (as determined by the pathologist), accurately classify DKK1 negative cells (as determined by the pathologist) as negative (false positive rate ≤ 20%) and classify tumor compartments and stromal compartments of the tissue. Sensitivity was defined as the ability of the algorithm to accurately identify cells when present (as determined by the pathologist) and accurately classify DKK1 positive cells (as determined by the pathologist) as positive (false negative rate ≤ 20%). Accuracy required a positive Pearson correlation with a statistically significant *p* value (*p* ≤ 0.05) between the manual DKK1 H-scores and digital DKK1 H-scores. For precision, the same acceptance criteria were applied as for the DKK1 RNAscope assay.

## Supplementary Information

Supplementary Figures

Supplementary Table S1

Supplementary Table S2

Supplementary Table S3

Supplementary Table S4

Supplementary Table S5

Supplementary Table S6
