# Supplementary material for: Validation of a DKK1 RNAscope chromogenic in situ hybridization assay for gastric and gastroesophageal junction adenocarcinoma tumors
Source: Sci Rep. 2021 May 10;11:9920. doi: 10.1038/s41598-021-89060-3 (PMC8110580; doi:10.1038/s41598-021-89060-3)
Supplement: Supplementary file 1 — Supplementary Figures [file 41598_2021_89060_MOESM1_ESM.pdf]

Supplementary Figures

Validation of a DKK1 RNAscope Chromogenic In Situ Hybridization Assay for Gastric and Gastroesophageal Junction Adenocarcinoma Tumors

Charles Caldwell, James B. Rottman, Will Paces, Elizabeth Bueche, Sofia Reitsma, Joseph Gibb, Vitria Adisetiyo, Michael S. Haas, Heidi Heath, Walter Newman, Jason Baum, Roberto Gianani, Michael H. Kagey

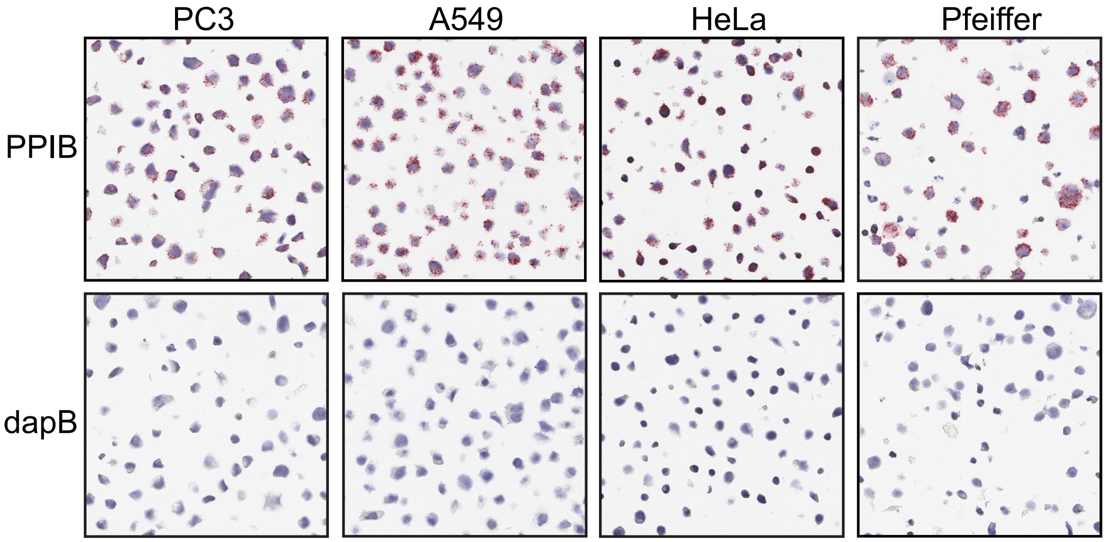

Supplementary Figure S1. PPIB and dapB RNAscope staining of the control CPA
A FFPE cell pellet array containing the indicated cell lines was evaluated for RNA integrity and background signal by RNAscope using probes to the moderately expressed housekeeping gene PPIB and the bacterial gene dapB. Cell pellet array (CPA).

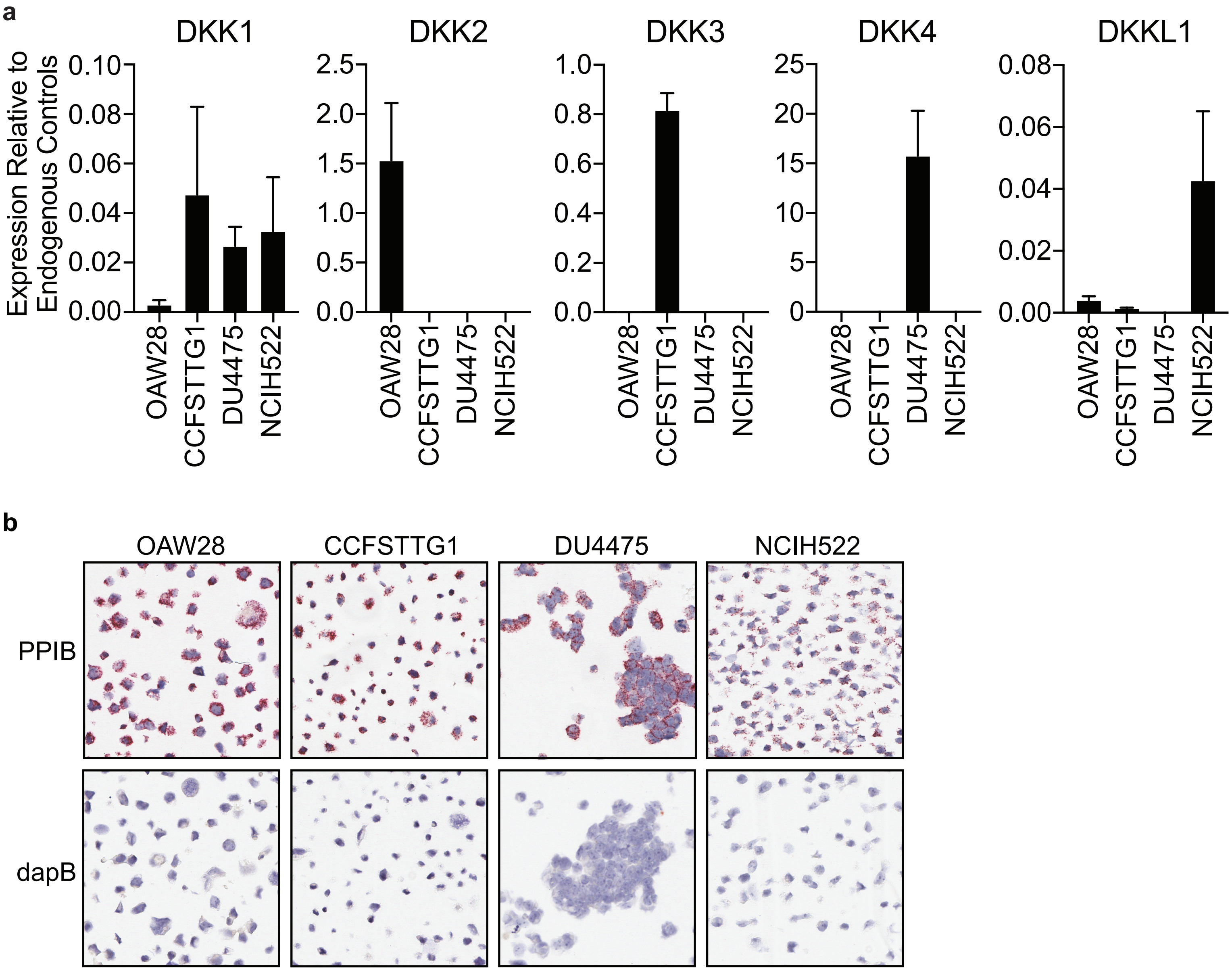

Supplementary Figure S2. Characterization of the Dickkopf family member specificity CPA
(**a**) Expression of Dickkopf family members from the indicated cell lines in the specificity CPA. qPCR data was normalized to GAPDH, TBP and SDHA housekeeping genes. (**b**) The FFPE specificity CPA containing the indicated cell lines was evaluated for RNA integrity and background signal by RNAscope using probes to the moderately expressed housekeeping gene PPIB and the bacterial gene dapB. Cell pellet array (CPA).

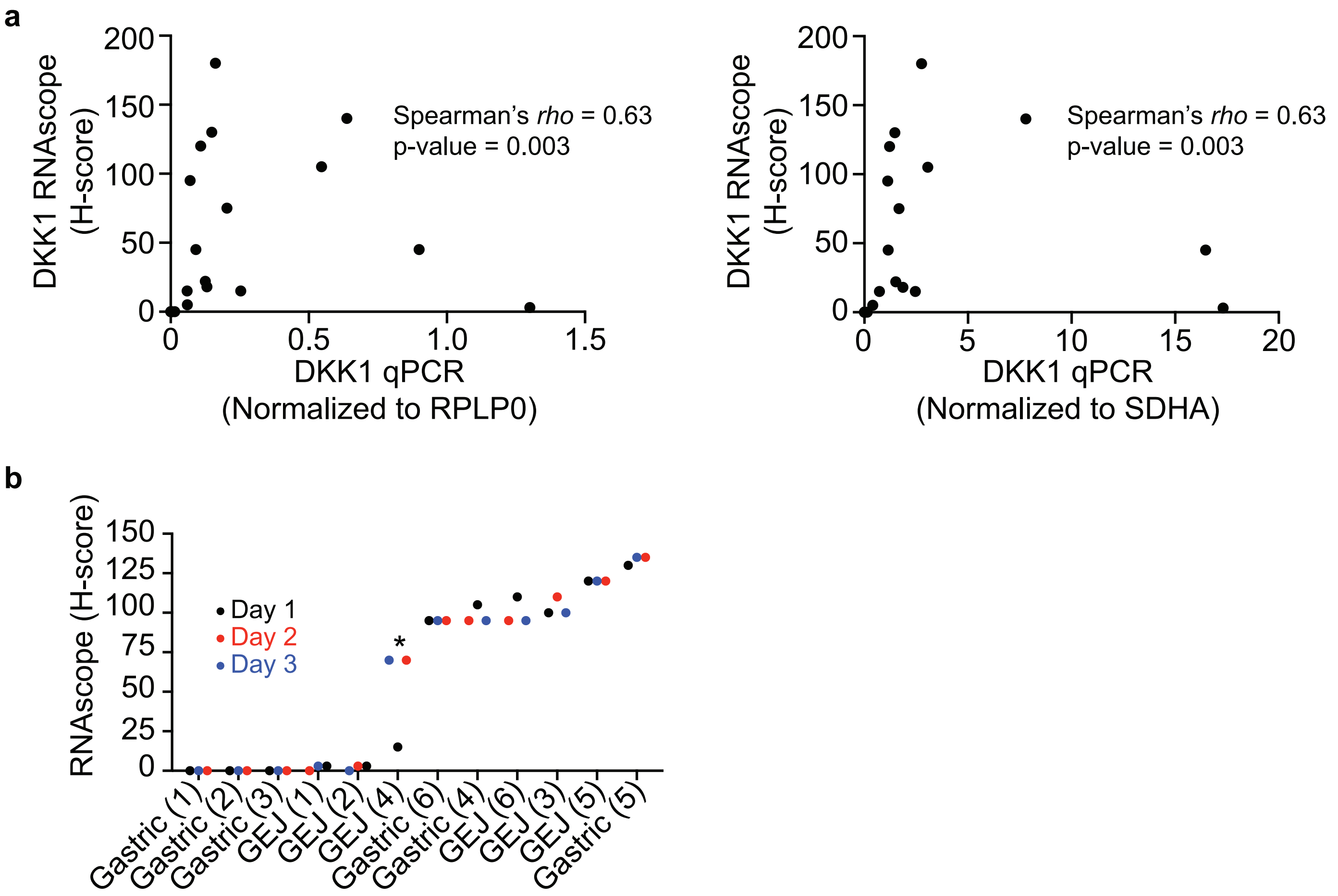

Supplementary Figure S3. Validation of accuracy and precision of the DKK1 RNAscope assay (**a**) Manual DKK1 RNAscope H-scores were compared to DKK1 qPCR results from tumor resections. Prior to RNA isolation for qPCR, regions with tumor cells were dissected. qPCR data was normalized to either RPLP0 (left graph) or SDHA (right graph) housekeeping genes. (**b**) Replicate DKK1 staining was conducted on 3 different days and manual H-scores were determined. All replicates were required to be in the same bin category or within an H-score of ±20 if discordant binning occurred. Bins were defined as, negative signal (H-score = 0), low signal (H-score < 34), and high signal (H-score ≥ 35). Asterisk indicates the one tumor resection that failed precision.

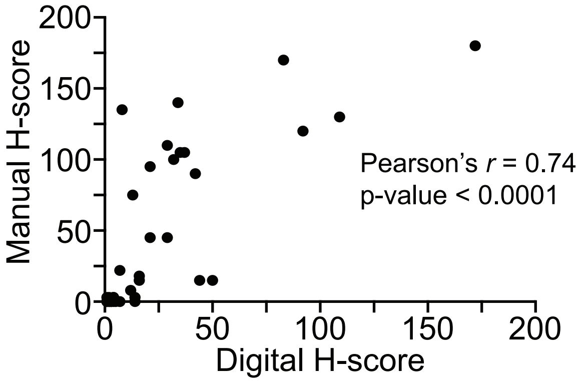

Supplementary Figure S4. Validation of the accuracy of the digital image analysis algorithm
Correlation of manual and digital H-scores from the tumor resections (n=36). The 4 tumor resections that did not pass the specificity assessment for the digital image analysis algorithm were excluded from the analysis.
